# Elevated lipid oxidation is associated with exceeding gestational weight gain recommendations and increased neonatal anthropometrics: a cross-sectional analysis

**DOI:** 10.1186/s12884-021-04053-4

**Published:** 2021-08-21

**Authors:** Jill M. Maples, Samantha F. Ehrlich, Nikki B. Zite, Kevin J. Pearson, W. Todd Cade, Courtney J. Riedinger, Maire M. Blankenship, Rachel A. Tinius

**Affiliations:** 1 Department of Obstetrics and Gynecology, University of Tennessee Graduate School of Medicine, 1924 Alcoa Highway, U-27, TN 37920 Knoxville, USA; 2 Department of Public Health, University of Tennessee, 37920 Knoxville, TN USA; 3 Department of Pharmacology and Nutritional Sciences, University of Kentucky College of Medicine, 40536 Lexington, Kentucky USA; 4 Doctor of Physical Therapy Division, Duke University School of Medicine, 27710 Durham, NC USA; 5 School of Nursing and Allied Health, Western Kentucky University, 42101 Bowling Green, KY USA; 6 School of Kinesiology, Recreation, and Sport, Western Kentucky University, 42101 Bowling Green, KY USA

**Keywords:** Birthweight, Gestational weight gain, Lipid metabolism, Lipid oxidation, Neonatal anthropometrics, Neonatal fat mass, Pregnancy

## Abstract

**Background:**

Deviations from gestational weight gain (GWG) recommendations are associated with unfavorable maternal and neonatal outcomes. There is a need to understand how maternal substrate metabolism, independent of weight status, may contribute to GWG and neonatal outcomes. The purpose of this study was to explore the potential link between maternal lipid oxidation rate, GWG, and neonatal anthropometric outcomes.

**Methods:**

Women (*N* = 32) with a lean pre-pregnancy BMI were recruited during late pregnancy and substrate metabolism was assessed using indirect calorimetry, before and after consumption of a high-fat meal. GWG was categorized as follows: inadequate, adequate, or excess. Shortly after delivery (within 48 h), neonatal anthropometrics were obtained.

**Results:**

Using ANOVA, we found that fasting maternal lipid oxidation rate (grams/minute) was higher (*p* = 0.003) among women with excess GWG (0.1019 ± 0.0416) compared to women without excess GWG (inadequate = 0.0586 ± 0.0273, adequate = 0.0569 ± 0.0238). Findings were similar when lipid oxidation was assessed post-meal and also when expressed relative to kilograms of fat free mass. Absolute GWG was positively correlated to absolute lipid oxidation expressed in grams/minute at baseline (r = 0.507, *p* = 0.003), 2 h post-meal (r = 0.531, *p* = 0.002), and 4 h post-meal (r = 0.546, *p* = 0.001). Fasting and post-meal lipid oxidation (grams/minute) were positively correlated to neonatal birthweight (fasting r = 0.426, *p* = 0.015; 2-hour r = 0.393, *p* = 0.026; 4-hour r = 0.540, p = 0.001) and also to neonatal absolute fat mass (fasting r = 0.493, *p* = 0.004; 2-hour r = 0.450, p = 0.010; 4-hour r = 0.552, *p* = 0.001).

**Conclusions:**

A better understanding of the metabolic profile of women during pregnancy may be critical in truly understanding a woman’s risk of GWG outside the recommendations. GWG counseling during prenatal care may need to be tailored to women based not just on their weight status, but other metabolic characteristics.

## Background

Deviations from the guidelines for gestational weight gain (GWG) from the US National Academy of Medicine (NAM; previously known as the Institute of Medicine) have been associated with unfavorable maternal and infant outcomes [1]. For example, exceeding the GWG recommendations during pregnancy increases the risk of many adverse maternal outcomes [2–4] including hypertensive disorders during pregnancy [5, 6], non-elective surgical deliveries [7, 8], and postpartum weight retention [3]. Adverse neonatal outcomes associated with excessive GWG are linked with excessive fetal growth and adiposity, which beyond the immediate risk of birth trauma, have implications for future risk of obesity and metabolic disease [9].

Because 70 % of women do not achieve GWG within recommended ranges set forth by NAM [10], a greater understanding of maternal metabolic factors that may influence excess GWG is warranted. Prior work has focused on the impact that pre-pregnancy weight status has on the risk of exceeding GWG recommendations and there is a general consensus that women with overweight/obesity are at greater risk of excessive GWG. Work by Bugatto et al. suggests women with excess adiposity during pregnancy oxidize substrates (i.e. lipids and carbohydrates) differently than lean women who are pregnant [11]. Specifically, lipid oxidation during late pregnancy is significantly higher among women that were overweight, compared to lean pregnant women. This is consistent with other reports that pregnant women with overweight/obesity are resistant to insulin-mediated suppression of lipid oxidation and lipolysis [12, 13]. In this scenario, the lack of insulin-mediated suppression of lipid metabolic processes could produce excess free fatty acids that could eventually be stored and contribute to excessive GWG. However, little is known about the potential impact that maternal lipid oxidation during pregnancy may have on exceeding GWG recommendations.

In addition, maternal substrate metabolism may contribute to infant health outcomes. Indicators of maternal lipid metabolism, such as maternal blood lipid profiles, have been consistently associated with neonatal birthweight and adiposity [14]. Diderholm et al. evaluated the impact that maternal lipolysis during late pregnancy had on fetal growth outcomes and found that lipolysis during late pregnancy was independently associated with fetal size [15]. The authors suggest that elevated lipolysis during late pregnancy could expose the fetus to excess substrates, other than glucose, which has historically been considered one of the most significant drivers of fetal growth [15]. These relationships provide some insight into maternal metabolism’s impact on neonatal outcomes; however, limited research has evaluated the impact of maternal lipid oxidation during late pregnancy on fetal growth and adiposity. Understanding factors that contribute to fetal overgrowth are important as macrosomia can have direct negative impacts on obstetric outcomes. For example, the risks of postpartum hemorrhage, chorioamnionitis, and more severe vaginal lacerations are elevated with macrosomia [16].

The purpose of this study was to explore the potential link between maternal lipid oxidation, GWG, and neonatal anthropometric outcomes. Specifically, maternal lipid oxidation rates during late pregnancy across women with a lean pre-pregnancy BMI were stratified by the NAM GWG recommendations (i.e. inadequate, adequate, excess) and compared. Potential relationships between maternal lipid oxidation, absolute GWG, and neonatal anthropometric outcomes were also explored. Because maternal weight status is an important factor determining fetal growth and adiposity, we sought to explore the potential impact of maternal lipid oxidation on neonatal anthropometric outcomes among exclusively lean women. Studying only normoglycemic, lean pregnant women adds to the novelty of the project as maternal weight status prior to pregnancy and clinically diagnosed insulin resistance during pregnancy will not confound relationships between GWG and outcomes.

## Methods

This is a cross-sectional study design that took place at a single academic institution. All study procedures were approved by the Western Kentucky University’s Institutional Review Board (IRB: 16–229, NCT: NCT03504319). All methods were performed in accordance with the Code of Federal Regulations on the Protection of Human Subject (45 CFR Part 46) and institutional research policies and procedures. All participants were informed of the benefits and risks of study participation prior to providing written informed consent.

### Participants

Participants were a subset from a larger previously published cohort [17]. In the parent study, participants were enrolled to determine the impact that overweight/obesity has on substrate metabolism in response to a high-fat meal challenge in late pregnancy. Participants were included if they were 18–44 years old, had a confirmed singleton viable pregnancy with no fetal abnormalities, and had obstetric provider release to participate in the study. Participants were excluded if they had multiple gestation pregnancy, currently smoked or used illicit drugs, or had a history of or current gestational diabetes, pre-pregnancy diabetes or prior macrosomic infant. In the parent study women with a self-reported BMI between 18.5 and 45 kg/m^2^ were included. We found that pregnant women with overweight/obesity were less metabolically flexible (i.e. had a dampened ability to increase lipid oxidation in response to the high-fat meal) compared to women that entered pregnancy with a lean weight status. These findings suggest that women with a lean pre-pregnancy weight status had a more favorable metabolic profile. Because of this, and in an effort to avoid the potential impact that excess pre-gravid adiposity may have on lipid oxidation and GWG, only the lean cohort from the parent study was chosen for the current study. The subset consisted of lean pregnant women, with a pre-pregnancy BMI between 18.5 and 25 kg/m^2^. Including only women with a lean pre-pregnancy weight status allowed us to more clearly investigate the role of GWG on metabolic health during pregnancy, without the potential confounding factor of excess adiposity. Inclusion criteria included: Age 18–44, confirmed singleton viable pregnancy with no fetal abnormalities at routine 18–22 ultrasonography, plan to deliver at The Medical Center, completion of a standard of care gestational diabetes screen, and obstetric provider release to participate in the study procedures. Exclusion criteria included: multiple gestation pregnancy, inability to provide voluntary informed consent, current use of illegal drugs (cocaine, methamphetamine, opiates, etc.), current smoker who does not consent to cessation, current usage of daily medications by class: corticosteroids, anti-psychotics (known to alter insulin resistance and metabolic profiles), history of gestational diabetes, pre-pregnancy diabetes or prior macrosomic (> 4500 g) infant (each elevate the risk for gestational diabetes in the current pregnancy, or undiagnosed gestational diabetes), and dietary restrictions prohibiting them from consuming the standardized meal/high-fat load.

### Maternal metabolic study visit

The maternal metabolic study visit has been described previously [17]. Briefly, participants reported to the lab for the metabolic study visit in the morning after an overnight fast. The night before the study visit, they were provided with written instructions for preparing and consuming a standardized diet. The instructions were to consume a standardized 800 kilocalorie meal, consisting of approximately 50 % carbohydrate, 30 % fat, and 20 % protein, at 6pm the evening before the study visit. Upon arrival to the lab for the study visit, the participant’s weight, height, and vitals were taken. Body composition was measured using skinfold anthropometry at seven sites in triplicate using calipers (Harpenden Skinfolds Caliper, Baty International, United Kingdom) in order to determine maternal percent body fat. For each participant, average skin fold thickness measures were entered into a standardized equation that accounts for age as previously described [18], a technique that has been used during pregnancy in prior studies [19, 20].

Using the TrueOne Canopy Option and TrueOne Metabolic Cart (TrueOne 2400, Parvomedics, Sandy, UT), fasting metabolic measurements were assessed via indirect calorimetry for approximately 15 min. Resting metabolic rate, carbon dioxide production, and oxygen consumption were measured. Using carbon dioxide production and oxygen consumption rates, lipid and carbohydrate oxidation rates were calculated as previously described [21]. A baseline, fasting blood draw was obtained after the baseline resting metabolism measurement was made. Participants then consumed a standardized 1000-kcal meal within 20 min that was high in fat and similar in composition to previous studies [22, 23]. The high-fat meal consisted of approximately 55 % fat, 30 % carbohydrate, and 15 % protein. Additional postprandial metabolic measurements (indirect calorimetry) were taken 2 and 4 h after the high-fat meal was consumed.

### Neonatal study visit

Within 48 h of delivery, trained study team members met with participants in the hospital postpartum unit. Neonatal body composition (fat and lean mass) was measured by skin fold thickness using calipers at the patients’ bedside. Skin fold measures at four sites (triceps, subscapular, ilium, and thigh) were assessed in duplicate in accordance with previously described protocols [24]. If duplicate readings were not within 0.5 mm of each other, a third measure was taken, as is customary with neonatal skinfold assessments [24]. Birthweight, length, head circumference, and abdominal circumference was assessed by nursing staff immediately after delivery and recorded by the study team during the neonatal study visit. Absolute fat mass was determined from the equation derived by Aris et al. [25].

### Gestational weight gain outcome

Pre-pregnancy weight was self-reported and documented during the metabolic study visit. Final delivery weight was self-reported and documented during the neonatal study visit. Total gestational weight gain was calculated and categorized as follows: inadequate (below the NAM guidelines), adequate (adhered to the NAM guidelines), or excess (exceeded the NAM guidelines) based on the calculated maternal pre-pregnancy BMI.

### Statistical analyses

Normality of the distribution for each variable was tested using Kolmogorov-Smirnov tests. For categorical data, chi-square tests were used to assess differences between groups. For continuous data, ANOVA with post-hoc analyses or Kruskal-Wallis tests were used to assess differences between groups. Pearson product-moment correlation coefficients for normally distributed variables or Spearman’s rank-order correlation coefficient for non-normally distributed variables were used to assess the degree of the relationship between variables. Partial correlations were used to adjust for potential confounders (e.g. baby’s sex, maternal pre-pregnancy BMI, GWG). All data analyses were conducted using IBM SPSS Statistics Version 26 (Armonk, New York).

## Results

Thirty-two women met inclusion criteria. Participant characteristics are shown in Table 1.

**Table 1:** Characteristics of women stratified by gestational weight gain category^a^

Participants, *N*=32	Inadequate (*n*=7)Mean ± SD	Adequate (*n*=14)Mean ± SD	Excess (*n*=11)Mean ± SD	*p*-value
Gestational Weight Gain (kilograms)*	8.5±2.01	13.3±1.3	18.8±2.3†‡	<0.001*
Pre-pregnancy BMI*	21.2±1.5	22.0±1.7	23.0±1.5‡	0.035*
Age (years)	26.7±6.6	31.6±3.8	29.8±3.7	0.082
Gestation Age at Study Visit (weeks)	34.5±2.7	34.5±1.7	34.3±1.6	0.977
Gestation Age at Delivery (weeks)	40.6±1.2	39.7±1.1	39.3±1.2	0.079
Percent Body Fat at Study Visit (%)^b^*	19.4±5.1	19.4±3.4	23.2±3.2†‡	0.033*
Systolic Blood Pressure (mmHg)*	112.4±11.2	116.3±13.3	117.8±14.6	0.705
Diastolic Blood Pressure (mmHg)	69.1±7.8	70.9±13.0	74.3±8.0	0.564
Parity^c^	n (%)	n (%)	n (%)	0.201
*Primiparous*	6 (86%)	7 (50%)	5 (55%)	
* Multiparous*	13 (14%)	7 (50%)	6 (45%)	
Ethnicity^c^	n (%)	n (%)	n (%)	0.515
*Caucasian*	9 (100%)	13 (93%)	11 (100%)	
* Hispanic*	0 (0%)	1 (7%)	0 (0%)	
Education^c^	n (%)	n (%)	n (%)	0.147
* High School Graduate*	2 (29%)	0 (0%)	0 (0%)	
* Trade School *	0 (0%)	0 (0%)	1 (9%)	
* College Graduate*	2 (29%)	6 (43%)	4 (36%)	
* Post- Graduate Degree*	3 (42%)	8 (57%)	6 (55%)	
Cholesterol (mg/dL)	290.2±48.7	257.4±40.8	255.6±28.5	0.176
LDL (mg/dL)^b^	184.2±48.5	145.8±39.2	135.5±35.2	0.064
HDL (mg/dL)	61.1±19.4	76.8±22.2	71.4±23.3	0.365
Triglycerides (mg/dL)	225.2±78.3	173.8±50.7	231.5±85.4	0.107
Free Fatty Acids(meq/L)	0.48±0.12	0.38±0.14	0.42±0.13	0.283
Insulin (uU/mL)	9.5±4.9	9.3±4.6	8.8±3.4	0.943
Glucose (mg/dL)	77.5±7.4	81.7±6.1	80.5±8.3	0.496
HOMA-IR	1.8±1.0	1.9±1.0	1.8±0.8	0.967

^a^ Groups were compared using ANOVA with post-hoc analyses, unless specifically noted

^b^ Groups compared using Kruskal-Wallis tests

^c^Groups compared using chi-square analysis

*Significant difference between groups (main effect), *p*<0.05

†Excess GWG group was significantly different from adequate GWG, *p*<0.05

‡ Excess GWG group was significantly different from inadequate GWG, *p*<0.05

The mean age was 29.9 ± 4.7 years, 56 % were primiparous, 97.0 % were non-Hispanic white, and 90 % had completed a college education. There were no differences in race, parity, or education across GWG categories.

**Fig. 1:**
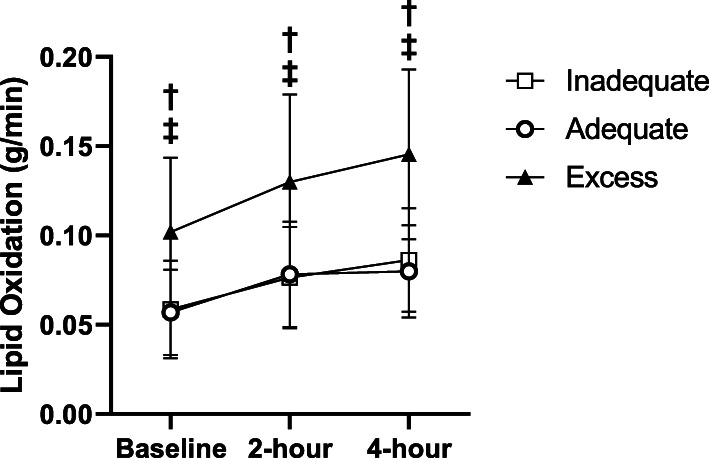
Maternal Lipid Oxidation

Lipid oxidation was higher among women that exceeded the GWG recommendation at baseline and in the postprandial states compared to women with adequate GWG (Table 2; Fig. 1). Consistent with this, respiratory quotient was lower among women with excessive GWG at all timepoints. Non-parametric methods of comparison revealed the same results.

**Table 2:** Maternal metabolic rate and substrate metabolism^a^

Participants, N = 32	Inadequate (*n* = 7)Mean ± SD	Adequate (*n* = 14)Mean ± SD	Excess (*n* = 11)Mean ± SD	*p*-value (95 % CI)
***Baseline/Fasted***				
Respiratory Quotient*	0.84 ± 0.07	0.85 ± 0.04	0.78 ± 0.05†	0.006* (0.034–0.491)
Resting Metabolic Rate per Kilogram Fat Free Mass (kilocalories/day)	27.27 ± 3.20	28.31 ± 4.29	31.97 ± 5.91	0.097 (0.000-0.354)
Lipid Oxidation (grams/minute)*	0.0586 ± 0.0273	0.0569 ± 0.0238	0.1019 ± 0.0416†‡	0.003* (0.051–0.515)
Lipid Oxidation per Kilogram Fat Free Mass (grams/minute)*	0.0012 ± 0.0006	0.0010 ± 0.0004	0.0017 ± 0.0007†	0.014* (0.012–0.460)
***Postprandial (2-hour)***				
Respiratory Quotient*	0.83 ± 0.07	0.83 ± 0.04	0.77 ± 0.05†	0.015* (0.011–0.448)
Resting Metabolic Rate per Kilogram Fat Free Mass (kilocalories/day)	34.31 ± 1.93	33.31 ± 4.66	37.38 ± 5.74	0.116 (0.000-0.342)
Lipid Oxidation (grams/minute)*	0.0764 ± 0.0284	0.0782 ± 0.0296	0.1300 ± 0.0490†‡	0.003* (0.052–0.516)
Lipid Oxidation per Kilogram Fat Free Mass (grams/minute)*	0.0014 ± 0.0007	0.0013 ± 0.0005	0.0021 ± 0.0008†	0.012* (0.015–0.466)
***Postprandial (4-hour)***				
Respiratory Quotient*	0.81 ± 0.06	0.83 ± 0.04	0.73 ± 0.05†‡	< 0.001* (0.160–0.620)
Resting Metabolic Rate per Kilogram Fat Free Mass (kilocalories/day)	33.47 ± 4.60	32.72 ± 6.51	35.40 ± 4.87	0.501 (0.000-0.212)
Lipid Oxidation (grams/minute)*	0.0863 ± 0.0291	0.0800 ± 0.0259	0.1454 ± 0.0475†‡	< 0.001* (0.0143–0.606)
Lipid Oxidation per Kilogram Fat Free Mass (grams/minute)*	0.0016 ± 0.0007	0.0014 ± 0.0004	0.0024 ± 0.0008†	0.002* (0.076–0.551)

^a^ Groups were compared using ANOVA with post-hoc analyses (because all data were normally distributed)

*Significant difference between groups (main effect), *p* < 0.05

†Excess GWG group was significantly different from adequate GWG, *p* < 0.05

‡ Excess GWG group was significantly different from inadequate GWG, *p* < 0.05

Absolute GWG was positively correlated to absolute lipid oxidation expressed in grams/min at baseline (R= 0.507, P= 0.003), 2 hours post-meal (R= 0.531, *P*=0.002), and 4 hours post-meal (R= 0.546, *P*= 0.001). Absolute GWG was also positively correlated to lipid oxidation relative to kg fat free mass at baseline (R= 0.396, *P*= 0.027), 2 hours post-meal (R= 0.456, *P*=0.010), and 4 hours post-meal (R= 0.456, *P*= 0.010).

**Fig. 2:**
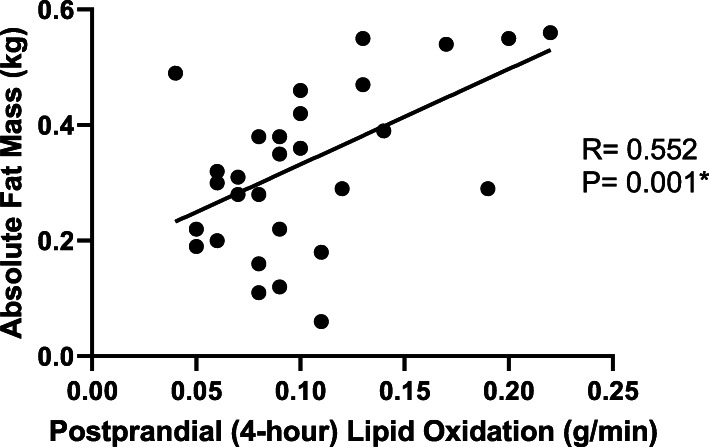
Maternal Lipid Oxidation and Infant Fat Mass

The average gestational age for the cohort was 39.7 ± 1.2 weeks, which was not different between maternal GWG groups (Table 1). The average birthweight for the cohort was 3400 ± 415 g, ranging from 2670 to 4090 g. There were no differences in neonatal anthropometric outcomes (birthweight, measures of adiposity, birth length, abdominal circumference) when stratified by maternal GWG groups (data not shown). However, maternal lipid oxidation was positively correlated to infant birthweight, total skin fold measures, and absolute fat mass (Fig. 2; Table 3).

**Table 3:** Relationship between maternal lipid oxidation and infant anthropometrics (*N*=32)

**Lipid Oxidation (grams/minute)**	**Baseline **	**2-Hour **	**4-Hour **
**Birthweight (grams) **	R= 0.426 P= 0.015*	R= 0.393 P= 0.026*	R= 0.540 P= 0.001*
**Total Skin Folds (millimeters)**	R= 0.380 P= 0.032*	R= 0.243 P= 0.181	R= 0.362 P= 0.042*
**Absolute Fat Mass (kilograms)** [25]	R= 0.493 P= 0.004*	R= 0.450 P= 0.010*	R= 0.552 P= 0.001*
**Lipid Oxidation per Kilogram Fat Free Mass (grams/minute)**	**Baseline **	**2-Hour **	**4-Hour **
**Birthweight (grams) **	R= 0.388 P= 0.031*	R= 0.365 P= 0.044*	R= 0.502 P= 0.004*
**Total Skin Folds (millimeters)**	R= 0.428 P= 0.016*	R= 0.295 P= 0.107	R= 0.413 P= 0.021*
**Absolute Fat Mass (kilograms) ** [25]	R= 0.450 P= 0.011*	R= 0.418 P= 0.019*	R= 0.510 P= 0.003*

*Significant correlation *p*<0.05

Values were similar when controlling for baby’s sex. Maternal lipid oxidation per kg fat free mass at 4 hours post-meal was positively correlated to birthweight (r=0.393, *p*=0.0326), total skinfolds (r=0.383, *p*=0.037) and absolute fat mass (r=0.381, *p*=0.038) even after controlling for GWG and pre-pregnancy BMI. These values were similar when maternal lipid oxidation was expressed in grams/min.

## Discussion

In the present study, we investigated the relationship between meeting the GWG recommendations and maternal lipid oxidation among lean women. The main findings from this study are that lipid oxidation is elevated during late pregnancy among women that were lean pre-pregnancy and exceed GWG recommendations, and that maternal lipid oxidation is positively correlated to baby weight and indicators of baby fat mass, even after controlling for absolute GWG. The results from this study indicate that maternal metabolic factors at the end of pregnancy, specifically lipid oxidation in the fasted state and in the postprandial state, may play an important role in GWG and neonatal weight and fat mass. It is possible that, even among pregnant women with a lean pre-pregnancy BMI, higher lipid oxidation in late pregnancy could predispose both mother and neonate to unfavorable metabolic consequences.

We found that lipid oxidation rates, in both the fasting and postprandial states, were elevated among pregnant women with a lean pre-pregnancy BMI that went on to exceed GWG recommendations compared to pregnant women with a lean pre-pregnancy BMI who met guidelines. (Fig. 1). A previous report described an increased reliance on carbohydrate metabolism during late pregnancy among normal weight, metabolically healthy pregnant women [26]. However, a recent study by Bugatto et al. found that pregnant women that are overweight have an increased reliance on lipid metabolism earlier in pregnancy and markedly higher lipid metabolism during late pregnancy compared to their lean counterparts [11]. Their study does not report measures of GWG among participants. It is possible lipid metabolism among the women in the current study with excessive GWG experienced a shift in substrate metabolism favoring lipid oxidation earlier in their pregnancy, contributing directly to the excess accumulation of maternal fat deposition and GWG.

Previous work among non-pregnant women has suggested that fat mass gain can result in increasing lipid oxidation rates, but that other factors beyond fat mass gain, account for the individual variability in lipid oxidation observed among women [27]. Given the cross-sectional design of the study, it is impossible to determine if the elevated lipid oxidation contributed to the GWG or rather if the excessive GWG caused the increase in lipid oxidation. Regardless of the order of events, lean women exceeding the GWG recommendations in the current study had elevated lipid oxidation in late pregnancy which is similar to the lipid oxidation profile among the overweight women in the Bugatto el al study [11]. To our knowledge, this study is the first study to provide evidence that elevated lipid oxidation among lean women with excessive GWG could be contributing to an unfavorable metabolic profile similar to pregnant women with pre-pregnancy overweight/obesity.

In addition to the implications of unfavorable metabolic adaptations on the mother, the neonates also appear to be impacted by maternal substrate oxidation as maternal lipid oxidation was related to infant adiposity (Fig. 2; Table 3). These findings confirm and elaborate on the idea that maternal substrate oxidation, beyond glucose, may play an important role in fetal growth and development [28–30]. A recent report by Diderholm et al. suggests lipolysis during late pregnancy is independently associated with fetal size [15]. The authors reported that, among pregnant women free of diabetes, 60 % of the variance in fetal weight estimated at 35 weeks gestation could be explained by maternal substrate metabolism. Specifically, they found that maternal rate of lipolysis was positively associated with fetal weight and found to be an independent predictor of fetal weight [15]. Another study by the same group found that impaired lipolysis in late pregnancy was associated with intrauterine growth restriction (IUGR) [31]. The authors suggest that reduced maternal substrate production, specifically lipolysis, could decrease the amount of substrate available for maternal oxidation and thereby cause a decrease in the amount of glucose available for the fetus.

Future studies exploring the impact of maternal substrate oxidation on GWG should consider recruiting women of varying weight statuses. In the current study we chose to include only women with a lean pre-pregnancy weight status as maternal overweight/obesity has been shown to be a risk factor for excessive GWG. Evidence suggests that lifestyle interventions, such as diet and exercise, can reduce the risk of excessive GWG among all pregnant women, including those with obesity [2]. It is possible that positive impact of lifestyle interventions on achieving adequate GWG is mediated by more favorable shifts in maternal substrate utilization.

This study is not without limitations. Objectively assessed GWG and serial measures of lipid oxidation and estimates of GWG throughout pregnancy, would have allowed a more rigorous exploration of the link between lipid oxidation and GWG. Assessing lipid oxidation and GWG throughout pregnancy would have allowed us to determine if women that exceeded the GWG recommendations had a relatively higher lipid oxidation early in pregnancy, in addition to during late pregnancy. Future work is needed to determine the clinical utility of assessing maternal substrate metabolism during pregnancy to predict those at increased risk of excessive GWG. Another limitation of this study was that the neonates included did not have extreme deviations from growth that is considered appropriate for gestational age. Therefore, we cannot determine if the positive correlation of maternal lipid oxidation to neonatal outcomes observed in this study would exist if neonates with more extreme variations in birthweight would have been included. Future studies should include pregnant women with small for gestational age and large for gestational age suspected fetuses. Also, this study was conducted among mostly highly educated, non-Hispanic white women. These findings may not be generalizable across more diverse populations and future studies should investigate the impact that maternal lipid oxidation has on gestational weight gain and neonatal outcomes among women from diverse racial and ethnic backgrounds.

## Conclusions

This study investigated the relationship between meeting the GWG recommendations and maternal lipid oxidation among lean women. Lipid oxidation is elevated during late pregnancy among women that were lean pre-pregnancy and exceed GWG recommendations. Additionally, maternal lipid oxidation was positively correlated to baby weight and indicators of baby fat mass, even after controlling for absolute GWG. Maternal metabolic factors at the end of pregnancy, specifically lipid oxidation in the fasted state and in the postprandial state, may play an important role in GWG and neonatal weight and fat mass. Higher lipid oxidation in late pregnancy could predispose both mother and neonate to unfavorable metabolic consequences, even among pregnant women with a lean pre-pregnancy weight status. A better understanding of the metabolic profile of women during pregnancy, including maternal lipid metabolism, could prove useful in terms of identifying women at risk for adverse pregnancy outcomes such as excessive GWG.

## Data Availability

The datasets generated during and/or analyzed during the current study are available from the corresponding author on reasonable request.

## References

[1] Voerman E, Santos S, Inskip H, Amiano P, Barros H, Charles MA (2019). Association of Gestational Weight Gain With Adverse Maternal and Infant Outcomes. Jama.

[2] Muktabhant B, Lawrie TA, Lumbiganon P, Laopaiboon M (2015). Diet or exercise, or both, for preventing excessive weight gain in pregnancy. Cochrane Database Syst Rev.

[3] Amorim AR, Rossner S, Neovius M, Lourenco PM, Linne Y (2007). Does excess pregnancy weight gain constitute a major risk for increasing long-term BMI?. Obesity (Silver Spring).

[4] Restall A, Taylor RS, Thompson JM, Flower D, Dekker GA, Kenny LC (2014). Risk factors for excessive gestational weight gain in a healthy, nulliparous cohort. J Obes.

[5] Chen Z, Du J, Shao L, Zheng L, Wu M, Ai M (2010). Prepregnancy body mass index, gestational weight gain, and pregnancy outcomes in China. Int J Gynaecol Obstet.

[6] Kiel DW, Dodson EA, Artal R, Boehmer TK, Leet TL (2007). Gestational weight gain and pregnancy outcomes in obese women: how much is enough?. Obstet Gynecol.

[7] Mamun AA, Callaway LK, O’Callaghan MJ, Williams GM, Najman JM, Alati R (2011). Associations of maternal pre-pregnancy obesity and excess pregnancy weight gains with adverse pregnancy outcomes and length of hospital stay. BMC Pregnancy Childbirth.

[8] Nohr EA, Vaeth M, Baker JL, Sorensen T, Olsen J, Rasmussen KM (2008). Combined associations of prepregnancy body mass index and gestational weight gain with the outcome of pregnancy. Am J Clin Nutr.

[9] Mamun AA, Mannan M, Doi SA (2014). Gestational weight gain in relation to offspring obesity over the life course: a systematic review and bias-adjusted meta-analysis. Obes Rev.

[10] Goldstein RF, Abell SK, Ranasinha S, Misso M, Boyle JA, Black MH (2017). Association of Gestational Weight Gain With Maternal and Infant Outcomes: A Systematic Review and Meta-analysis. Jama.

[11] Bugatto F, Quintero-Prado R, Vilar-Sánchez JM, Perdomo G, Torrejón R, Bartha JL (2017). Prepregnancy body mass index influences lipid oxidation rate during pregnancy. Acta Obstet Gynecol Scand.

[12] Cade WT, Tinius RA, Reeds DN, Patterson BW, Cahill AG (2016). Maternal Glucose and Fatty Acid Kinetics and Infant Birth Weight in Obese Women With Type 2 Diabetes. Diabetes.

[13] Sivan E, Homko CJ, Chen X, Reece EA, Boden G (1999). Effect of insulin on fat metabolism during and after normal pregnancy. Diabetes.

[14] Barbour LA, Hernandez TL (2018). Maternal Lipids and Fetal Overgrowth: Making Fat from Fat. Clin Ther.

[15] Diderholm B, Beardsall K, Murgatroyd P, Lees C, Gustafsson J, Dunger D (2017). Maternal rates of lipolysis and glucose production in late pregnancy are independently related to foetal weight. Clin Endocrinol (Oxf).

[16] Macrosomia, ACOG (2020). ACOG Practice Bulletin, Number 216. Obstet Gynecol.

[17] Tinius RA, Blankenship MM, Furgal KE, Cade WT, Pearson KJ, Rowland NS (2020). Metabolic flexibility is impaired in women who are pregnant and overweight/obese and related to insulin resistance and inflammation. Metabolism.

[18] Jackson AS, Pollock ML, Ward A (1980). Generalized equations for predicting body density of women. Medicine and science in sports and exercise.

[19] Kannieappan LM, Deussen AR, Grivell RM, Yelland L, Dodd JM (2013). Developing a tool for obtaining maternal skinfold thickness measurements and assessing inter-observer variability among pregnant women who are overweight and obese. BMC Pregnancy Childbirth.

[20] Taggart NR, Holliday RM, Billewicz WZ, Hytten FE, Thomson AM (1967). Changes in skinfolds during pregnancy. Br J Nutr.

[21] Frayn KN (1983). Calculation of substrate oxidation rates in vivo from gaseous exchange. Journal of applied physiology: respiratory, environmental and exercise physiology.

[22] Heilbronn LK, Gregersen S, Shirkhedkar D, Hu D, Campbell LV (2007). Impaired fat oxidation after a single high-fat meal in insulin-sensitive nondiabetic individuals with a family history of type 2 diabetes. Diabetes.

[23] Jakulj F, Zernicke K, Bacon SL, van Wielingen LE, Key BL, West SG (2007). A high-fat meal increases cardiovascular reactivity to psychological stress in healthy young adults. J Nutr.

[24] Cauble JS, Dewi M, Hull HR (2017). Validity of anthropometric equations to estimate infant fat mass at birth and in early infancy. BMC Pediatr.

[25] Aris IM, Soh SE, Tint MT, Liang S, Chinnadurai A, Saw SM (2013). Body fat in Singaporean infants: development of body fat prediction equations in Asian newborns. Eur J Clin Nutr.

[26] Butte NF, Hopkinson JM, Mehta N, Moon JK, Smith EO (1999). Adjustments in energy expenditure and substrate utilization during late pregnancy and lactation. Am J Clin Nutr.

[27] Schutz Y, Tremblay A, Weinsier RL, Nelson KM (1992). Role of fat oxidation in the long-term stabilization of body weight in obese women. Am J Clin Nutr.

[28] Hoet JJ, Hanson MA (1999). Intrauterine nutrition: its importance during critical periods for cardiovascular and endocrine development. J Physiol.

[29] Crume TL, Shapiro AL, Brinton JT, Glueck DH, Martinez M, Kohn M (2015). Maternal fuels and metabolic measures during pregnancy and neonatal body composition: the healthy start study. J Clin Endocrinol Metab.

[30] Kulkarni SR, Kumaran K, Rao SR, Chougule SD, Deokar TM, Bhalerao AJ (2013). Maternal lipids are as important as glucose for fetal growth: findings from the Pune Maternal Nutrition Study. Diabetes Care.

[31] Diderholm B, Stridsberg M, Nordén-Lindeberg S, Gustafsson J (2006). Decreased maternal lipolysis in intrauterine growth restriction in the third trimester. Bjog.

